# Original Communications

**Published:** 1851-08

**Authors:** 


### MEDICAL EXAMINER,

AND

### RECORD OF MEDICAL SCIENCE.

NEW SERIES.—NO. LXXX.—AUGUST, 1851.

### ORIGINAL COMMUNICATIONS.

To the Editors of the Medical Examiner:

GENTLEMEN:—I have extracted from my "case-book," and now place at your disposal, the history of a case of Tubercular Meningitis, which will serve to illustrate the obscurity which sometimes attends the commencement and early stages of this disease.

Yours truly,

FRANCIS WEST,

352 Chesnut St.

*Philada., July 20th,* 1851.

*Oct.* 24, 1837.—I was requested to-day to visit, for the Dispensary, a colored girl, aged 25 years, unmarried, living as a domestic in the family of ———, Lombard St. The patient was in the act of lighting the entry-lamp when I arrived. Upon examination, she complained chiefly of cough and pain in her chest beneath the clavicles, with some sore throat. Her complexion and general appearance were strongly indicative of the scrofulous diathesis, and led me to suspect the existence of pulmonary tubercles. An appropriate treatment was accordingly directed. Two days afterwards I saw her again; she was then in bed and complained of shooting pains through her head, particularly in the scalp, apparently neuralgic in character. Her pulmonary symptoms were better. The skin was but moderately warm; pulse not much excited; no sickness of stomach. Cups were ordered to the nape of the neck, with some laxative medicine. On the next day she expressed herself as relieved of the headache, but she now had all the ordinary symptoms of fever—dry tongue, thirst, hot skin, no appetite, &c. Neutral mixture was directed and a strict diet enjoined. In a day or two she was much better; tongue cleaning; skin cool and natural; pulse quite regular in every respect. During all my visits I had noticed in her an indisposition to communicate freely either with the family or myself, together with some somnolency at times, both of which conditions, however, were traced by the family to "natural sullenness." (This kind of taciturnity is very generally remarked as one of the characteristic symptoms of tubercular meningitis, and such is my own experience.)

On the 28th, at noon, four days after my first visit, my attendance was requested in haste, as the patient had suddenly become much worse. I found her almost insensible, with hot skin; pulse about 100, small and tense; tongue brown and dry; abdomen very painful, especially upon pressure, chiefly evinced by contortion of the countenance. Blisters were directed to her ankles, fomentations to the abdomen, with free cupping over same part, and cool drinks.

In the evening much the same; cups had not drawn well. At this time I was "halting between two opinions," not feeling perfectly assured whether the cerebral symptoms were primary or only sympathetic of abdominal disease. Small doses of calomel (gr. $\frac{1}{8}$) had been directed in consequence of the black-colored, tarry appearance of the stools. In the evening she was directed to be freely leeched to the temples and behind the ears, and to have purgative enemata administered. These latter brought away large quantities of black, tarry matter, the discharge of which was followed by such marked amelioration of the symptoms that I scarcely regretted the omission of the leeching, through negligence of the leecher.

*Oct.* 29.—Patient is much better this morning. Quite sensible, and has spoken to those about her so intelligently, as to induce a belief that the cerebral symptoms were the effect of an overloaded and unhealthy condition of the bowels. Injection repeated; tongue much cleaner and more moist; desired nourishment. Notwithstanding this apparent amendment in some of the symptoms, she nevertheless at times, evinced a good deal of somnolency and indisposition to mental exertion. This was attributed as before, in a great measure, to natural temperament, and did not materially abate the favorable prognosis drawn from the other circumstances mentioned. Light nourishment was allowed, and revulsion kept up internally, by purgatives, and externally, by sinapisms, applied to the lower extremities.

On the next day she sat up on the side of the bed for an hour and a half, her muscular power being but little impaired. During a few days back, she had had no fever, pulse but little above the natural standard, the only marked deviation from health indeed, exhibited by her, being occasional *somnolency*, and more or less constant *mental dulness and inertia.* At times, when spoken to, she replied without exact meaning to her words—her mouth is kept more or less constantly closed, and she manifests unwillingness and hesitancy about protruding her tongue. She is able to swallow perfectly, and with her own hands put some grapes into her mouth and ate them with apparent relish. Her eyes are rather vacant, but occasionally they *become fixed* upon the person speaking to her, betraying, however, consciousness of what is said. When asked, she does not complain of any head-ache; is able to sit up in bed against a chair without any fatigue; abdomen painfully sensible to pressure. The somnolency has slightly increased; head of perfectly natural temperature; general surface quite pleasant in regard to heat and moisture—pulse about 100 and strong; is unwilling to protrude the tongue; there is not, and never has been, the slightest disposition to convulsion or paralysis. Bowels open, with black discharges—no vomiting; during the whole attack her skin has been strikingly insensible to the action of blisters and sinapisms, none of which have produced any effect. Position in bed quite natural, but remains almost immoveably the same. Upon making an effort to arouse her by talking to her and gently slapping her chest, she turned and looked indifferently at me. She continued much in the same condition from this time until the 10th of the following month, (November.) Now and then the symptoms remitting a little, but upon the whole sensibly and gradually increasing. In consequence of sickness in the family, it became necessary to-day, Nov. 11th, to remove her from the house. The removal was effected as easily as possibly, but was followed by very great prostration, requiring the free use of active internal stimulants. On the 12th, two days after reaching her new quarters, she was suddenly attacked by paralysis of the whole left side of her body, and on the following day she died.

*Autopsy*—12 hours after death. Tubercles disseminated generally throughout the organs, the lungs being perfectly studded with them. They were miliary in character. The alimentary canal presented nothing remarkable—the peritoneum was slightly inflamed throughout, with depositions here and there, of minute tubercular granulations.

The brain, or rather its investing membranes, exhibited evident traces of the disease which had so insidiously commenced, and had been so obscurely marked throughout the great part of its course. The brain was slightly congested at several points, but was unchanged in consistence.

The membranes covering the upper portion or convexity, especially the pia mater, were very much injected and slightly thickened, with patches which were perfectly white and opaque, the whole being raised by a lactescent serous fluid effused beneath, which at the very summit gave the appearance of a distended sac. The same kind of lesion exactly was found at the base of the brain; the membranes at this point, and especially the pia mater covering the tubercula quadrigemina, enclosing a considerable deposit of purulent matter, mixed with tuberculous secretion in form of small granulations and shreds. Throughout the membranes themselves too, minute tubercular granulations were observable, resembling the appearances presented in the arachnitis of children.

In the opinion of all present at the autopsy, the disease must have been progressing for some time, and yet it failed to manifest itself at all, until about 18 days before the fatal result; and indeed, as before observed, the only *constant* symptoms of cerebral disturbance, were the sullen disposition and mental dulness! In more than one case of tubercular meningitis falling under my care, precisely the same condition has been remarked; and in one instance particularly, this kind of lesion manifested itself by what was believed to be an attack of acute mania.

In the present case the patient complained of pain for a short time only, and just after the attack commenced. This symptom is usually a very prominent one in tubercular meningitis. The abatement for a time of the cerebral disturbance, with the accession of the ordinary phenomena of continued fever, coupled with the almost entire disappearance of disease, after the free discharge from the bowels, were calculated to confirm the suspicion of its abdominal character; and to lead to some confusion of diagnosis. This not unfrequently happens in cases of tubercular meningitis, not very clearly marked, its proper symptoms being obscured by, and at times almost completely merged in those proceeding from sympathetic or primary disorder of other parts, and especially of the alimentary canal. This alternation of one class of symptoms with the other, makes it necessary for the physician to exercise special care and discrimination in fixing positively the *locale* of the disease, which is thus liable, as has been seen, to exhibit such uncertain manifestations. In the early stage of tubercular meningitis, and whenever more certain signs are wanting, the occurrence of continued somnolency, with dulness of intellect and a moody, sullen disposition, will materially assist in the detection of the disease, or at least should always lead to the suspicion of its existence. The external indications of a tubercular diathesis, together with the discovery, by physical signs, of the actual presence of tubercles in the lungs or other organs, will, of course, go far in corroborating our judgment upon this important point. At the period of making the present observation, the above mentioned aid to a correct diagnosis, unhappily, was not employed to the extent which all should now unite in recommending.

The history of the case may, perhaps, serve to put others, as it ever since has done myself, more and more upon their guard in treating obscure, or imperfectly marked tubercular meningitis.

